# Monthly Rates of Patients Who Left Before Accessing Care in US Emergency Departments, 2017-2021

**DOI:** 10.1001/jamanetworkopen.2022.33708

**Published:** 2022-09-30

**Authors:** Alexander T. Janke, Edward R. Melnick, Arjun K. Venkatesh

**Affiliations:** 1 Department of Emergency Medicine, Yale School of Medicine, New Haven Connecticut; 2 Institute for Healthcare Policy and Innovation, University of Michigan, Ann Arbor; 3 Now with VA Ann Arbor Healthcare System/University of Michigan, National Clinician Scholars Program, Ann Arbor; 4 Division of Health Informatics, Department of Biostatistics, Yale School of Public Health, New Haven, Connecticut; 5 Center for Outcomes Research and Evaluation, Yale School of Medicine, New Haven, Connecticut

## Abstract

This cross-sectional study investigates rates of patients who left emergency departments without being seen from 2017 to 2021.

## Introduction

Acute care demands have increased dramatically alongside stagnant hospital capacity,^1^ and extremes of emergency department (ED) boarding have become endemic.^2^ EDs are unique access points for comprehensive acute diagnostics and treatment in an otherwise-fragmented system.^3^ Patients often leave EDs before clinical evaluation (left without being seen [LWBS]) when EDs are crowded and wait times are long. These departures may have significant consequences for patients given the associated delayed or deferred care for acute conditions.^4^ Patients from minoritized communities are more likely to depart before evaluation, with problems relatively concentrated among hospitals serving low-income populations.^5^ This occurs despite the increasing added value of ED-based linkages to care for populations at increased risk, such as patients with opioid use disorder seeking medication treatment.^6^ We sought to characterize how often patients left the ED before clinical evaluation over time using a national sample of US hospitals.

## Methods

This cross-sectional study was classified as exempt from review and informed consent by the Yale University Institutional Review Board because it contained no patient data; all reporting adheres to STROBE reporting guidelines. The study used aggregated hospital measures available through a voluntary peer benchmarking service offered by Epic Systems Corporation, an electronic health record vendor. Measures were collected monthly from 2017 to 2021. Median (IQR) and 95th percentiles were reported for ED LWBS rates across hospitals each month. Statistical analyses were performed using R statistical software version 4.2.1 (R Project for Statistical Computing) from January to March 2022.

## Results

There were 365 hospitals reporting benchmarking data in January 2017, increasing to 1769 hospitals by December 2021. Annual ED visit volumes and total hospital beds for participating sites are included in the Table. Median (IQR) hospital LWBS rates nearly doubled from 1.1% (0.5%-2.5%) in 2017 to 2.1% (0.6%-4.6%) by the end of 2021 (Figure). Among the worst performing hospitals at the 95th percentile, 10.0% of ED patients left before a medical evaluation at the end of 2021, compared with 4.4% in the 95th percentile in January 2020 and 4.3% at the beginning of 2017.

**Table.** Site Characteristics for Participating Hospitals, 2021

Characteristic	Hospitals, No. (%) (N = 1769)
Hospital beds, No.	
<100	743 (42.0)
100-299	495 (28.0)
300-499	248 (14.0)
500-999	212 (12.0)
≥1000	71 (4.0)
Annual ED visits, No.	
0-20 000	513 (29.0)
20-40 000	531 (30.0)
40-60 000	371 (21.0)
60-80 000	177 (10.0)
≥80 000	177 (10.0)

Abbreviation: ED, emergency department.

**Figure.**
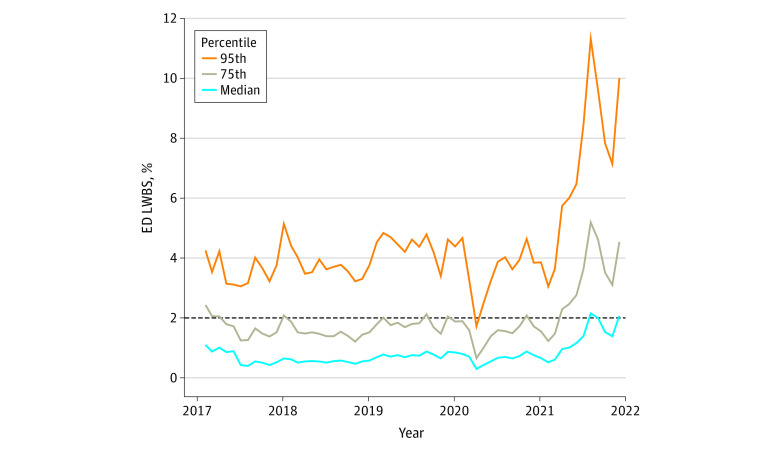
Left Without Being Seen (LWBS) Rates in Peer Benchmarking Service Peer benchmarking service data on monthly LWBS rates, inclusive of 365 hospital-based emergency departments (EDs) in 2017 and increasing to 1769 hospital-based EDs nationwide by the end of 2021, are presented.

## Discussion

Findings from this cross-sectional study demonstrate the failure of the emergency care system to maintain broad access in the context of pandemic demands, suggesting that existing regulatory and financial incentives may be inadequate to meet challenges of future pandemic waves and other disasters. Historically, LWBS was viewed as an ED management problem rather than a hospital- or systems-level issue. Thus, most solutions to date have relied on intradepartmental operational fixes to mitigate ED crowding; for example, doctor-in-triage or split-flow models offer more rapid medical screening evaluations, effectively bypassing traditional triage processes. These processes promote rapid but limited physician evaluations, often in the waiting room. Amid the current crisis, these ED-focused operational efforts may be inadequate to stem this growing problem.

Our work is observational, and due to limitations of available data fields, we were not able to address hospital characteristics or local COVID-19 infections or address how the mix of participating hospitals changed over time. We hypothesize that system strain would be associated with increased differences in rates of LWBS for hospitals serving low-income and underinsured patient populations. Furthermore, while some work addresses the association of LWBS with patient outcomes, there is little contemporaneous work on this for patients during COVID-19.

Access to emergency care cannot be considered universal until all patients presenting to EDs receive high-quality treatment for time-sensitive conditions. Given contributing system constraints, LWBS should be viewed as a failure to offer equitable access to acute care, understood in the context of other measures of care access.
